# ARTC1-mediated VAPB ADP-ribosylation regulates calcium homeostasis

**DOI:** 10.1093/jmcb/mjad043

**Published:** 2023-06-28

**Authors:** Xueyao Ma, Mengyuan Li, Yi Liu, Xuefang Zhang, Xiaoyun Yang, Yun Wang, Yipeng Li, Jiayue Wang, Xiuhua Liu, Zhenzhen Yan, Xiaochun Yu, Chen Wu

**Affiliations:** College of Life Sciences, Institute of Life Sciences and Green Development, Hebei University, Baoding 071002, China; College of Life Sciences, Institute of Life Sciences and Green Development, Hebei University, Baoding 071002, China; College of Life Sciences, Institute of Life Sciences and Green Development, Hebei University, Baoding 071002, China; College of Life Sciences, Institute of Life Sciences and Green Development, Hebei University, Baoding 071002, China; College of Life Sciences, Institute of Life Sciences and Green Development, Hebei University, Baoding 071002, China; Department of Public Health, Affiliated Hospital of Hebei University, Baoding 071000, China; College of Life Sciences, Institute of Life Sciences and Green Development, Hebei University, Baoding 071002, China; College of Life Sciences, Institute of Life Sciences and Green Development, Hebei University, Baoding 071002, China; College of Life Sciences, Institute of Life Sciences and Green Development, Hebei University, Baoding 071002, China; College of Life Sciences, Institute of Life Sciences and Green Development, Hebei University, Baoding 071002, China; Westlake Laboratory of Life Sciences and Biomedicine, Hangzhou 310024, China; School of Life Sciences, Westlake University, Hangzhou 310024, China; Institute of Biology, Westlake Institute for Advanced Study, Hangzhou 310024, China; College of Life Sciences, Institute of Life Sciences and Green Development, Hebei University, Baoding 071002, China

**Keywords:** ATRCs, mono-ADP-ribosylation, hARTC1, VAPB, calcium homeostasis

## Abstract

Mono-ADP-ribosylation (MARylation) is a post-translational modification that regulates a variety of biological processes, including DNA damage repair, cell proliferation, metabolism, and stress and immune responses. In mammals, MARylation is mainly catalyzed by ADP-ribosyltransferases (ARTs), which consist of two groups: ART cholera toxin-like (ARTCs) and ART diphtheria toxin-like (ARTDs, also known as PARPs). The human ARTC (hARTC) family is composed of four members: two active mono-ADP-ARTs (hARTC1 and hARTC5) and two enzymatically inactive enzymes (hARTC3 and hARTC4). In this study, we systematically examined the homology, expression, and localization pattern of the hARTC family, with a particular focus on hARTC1. Our results showed that hARTC3 interacted with hARTC1 and promoted the enzymatic activity of hARTC1 by stabilizing hARTC1. We also identified vesicle-associated membrane protein-associated protein B (VAPB) as a new target of hARTC1 and pinpointed Arg50 of VAPB as the ADP-ribosylation site. Furthermore, we demonstrated that knockdown of *hARTC1* impaired intracellular calcium homeostasis, highlighting the functional importance of hARTC1-mediated VAPB Arg50 ADP-ribosylation in regulating calcium homeostasis. In summary, our study identified a new target of hARTC1 in the endoplasmic reticulum and suggested that ARTC1 plays a role in regulating calcium signaling.

## Introduction

Protein ADP-ribosylation is a highly conserved and reversible post-translational modification that utilizes nicotinamide adenine dinucleotide (NAD^+^) as a donor and covalently attaches the ADP-ribose (ADPr) moiety onto target proteins. The enzymes that catalyze ADP-ribosylation are known as ADP-ribosyltransferases (ARTs), which are found in organisms ranging from prokaryotes to mammals. In mammals, ADP-ribosylation regulates various cellular and biological processes, including DNA damage repair, metabolism, and stress and immune responses. ADP-ribosylation can be categorized into two subtypes: mono-ADP-ribosylation (MARylation) and poly-ADP-ribosylation (PARylation) (Hottiger, 2015).

MARylation has been shown to be important in mitochondrial activity, stress responses, intracellular trafficking, and cell signaling (Di Girolamo et al., 1995; Haigis et al., 2006; Dani et al., 2011, 2013; Leung et al., 2011; Scarpa et al., 2013). The human ART cholera toxin-like (hARTC) family contains four members: two active mono-ARTs (hARTC1 and hARTC5) and two enzymatically inactive enzymes (hARTC3 and hARTC4) (Glowacki et al., 2002). hARTC1, hARTC3, and hARTC4 have a stretch of hydrophobic amino acids at their C-terminus, typical of glycosylphosphatidylinositol (GPI)-anchored membrane proteins. hARTC5, however, lacks this hydrophobic region and is an extracellular secreted enzyme (Okazaki and Moss, 1999; Glowacki et al., 2002; Stilla et al., 2011). hARTC1 and hARTC5 have been characterized as arginine-specific mono-ARTCs. The catalytic domain of the ARTCs is encoded by a single exon present in all ARTCs, and it contains an Arg–Ser–Glu (R–S–E) triad, with the conserved Glu being crucial for catalytic activity. The motif R–S–EXE is typical of these arginine-specific ARTCs. However, ARTC3 and ARTC4 lack this motif and thus have no ART activity (Glowacki et al., 2002).

ARTC1 is the first identified arginine-specific ART in mammals, initially isolated and characterized from rabbit skeletal muscle microsomal membranes (Peterson et al., 1990; Zolkiewska et al., 1992). Subsequent studies showed that ARTC1 was highly expressed in cardiac and skeletal muscles and conserved among different species, such as the human, rabbit, and mouse (Okazaki et al., 1996a, b). hARTC1 is expressed at the cell surface and localized in the endoplasmic reticulum (ER) (Fabrizio et al., 2015). Substrates of ARTC1 have been extensively investigated. Recently, Leutert et al. (2018) analyzed mouse ARTC1 (mARTC1)-specific ADP-ribosylome in C2C12 myotubes as well as skeletal and heart muscles of wild-type and ARTC1-deficient mice. The comprehensive systems-level analysis revealed that mARTC1 is essential for the modification of numerous proteins, providing strong evidence for the physiological importance of mARTC1. The authors found that hemopexin (HPX), the strongest heme-binding protein, is a direct mARTC1 target. The ADP-ribosylation of HPX by mARTC1 impaired its heme binding capacity and might therefore be a physiologically relevant function (Leutert et al., 2018). hARTC3 and hARTC4 have lost arginine-specific enzymatic activity, and their functions remain unknown. hARTC5 could auto-ADP-ribosylate itself and might be responsible for protein ADP-ribosylation in the extracellular environment, but reports about its protein substrates remain relatively scarce (Glowacki et al., 2002).

Vesicle-associated membrane protein-associated protein B (VAPB) is an integral ER membrane protein containing a C-terminal transmembrane domain inserted into the ER membrane and a cytoplasmic N-terminal tail with a coiled-coil domain and a major sperm protein (MSP) domain. Modulating the expression of VAPB affects Ca^2+^ exchange between the ER and mitochondria, which is a physiological readout of ER–mitochondria contacts (De Vos et al., 2012; Stoica et al., 2014). VAPB acts as a key player in facilitating tight membrane contact sites between the ER and other intracellular membranes, representing functional interaction through which Ca^2+^ exchange and lipid transfer occur (Muallem et al., 2017; Wu et al., 2018). VAPB is directly associated with amyotrophic lateral sclerosis (ALS) and regulates calcium homeostasis in ALS (De Vos et al., 2012). Lindhout et al. (2019) reported that the interaction of VAPB and secernin-1 (SCRN1) controls dynamic ER remodeling and, consequently, calcium homeostasis and synaptic vesicle cycling. The MSP domain of VAPB exhibits a FFAT-like binding site, and VAPB interacts with SCRN1 at the ER membrane via a single FFAT-like motif of SCRN1 (Lindhout et al., 2019).

Despite recent discoveries, the substrates of different hARTCs and their implications for cellular processes are not yet well understood. Here, we systematically analyzed the phylogenetic relatedness of the ARTC family, as well as the expression and localization patterns, with a particular focus on hARTC1. We found that knockdown of *hARTC1* impaired intracellular calcium homeostasis in human cardiomyocyte cells. Furthermore, our study revealed that hARTC3 and hARTC1 formed a heterodimer that enhanced the enzymatic activity of hARTC1 by stabilizing it. We also found that hARTC1 could ADP-ribosylate VAPB at Arg50, thereby facilitating the interaction between VAPB and SCRN1. Thus, we propose that hARTC1 plays a crucial role in ADP-ribosylating VAPB and modulates the biological activities of VAPB in calcium homeostasis.

## Results

### Phylogenetic relationship, motif, and homology of ARTC proteins

**Figure 1:**
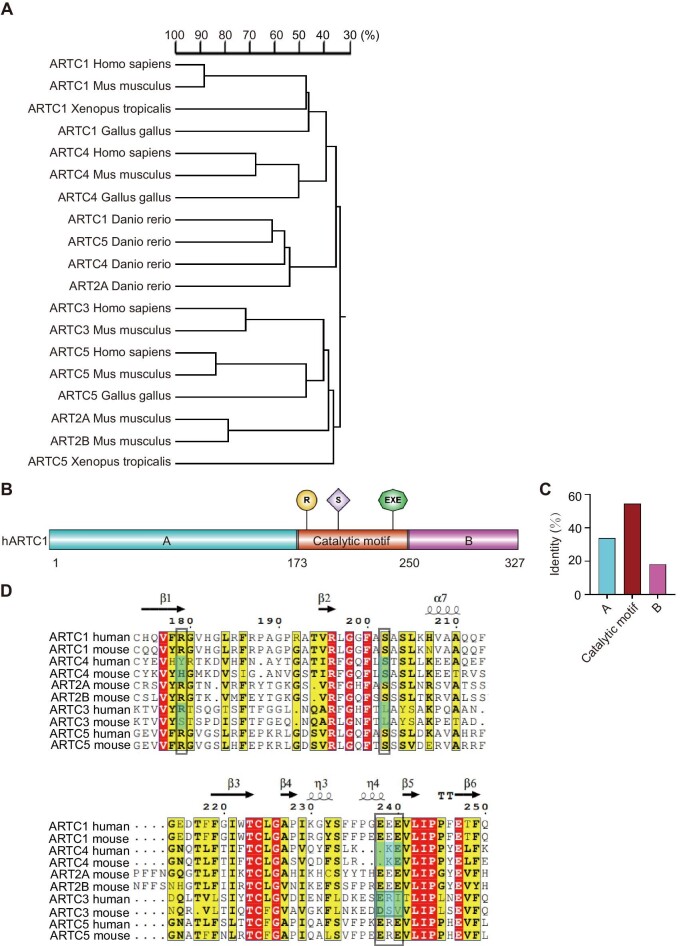
The phylogenetic relationship, conserved protein motifs, and homology of ARTCs. (**A**) A phylogenetic tree of six ARTC proteins in five representative species. The unrooted NJ phylogenetic tree was constructed using MEGA 6, and the ruler represents the percentage of homologous sequences. (**B**) Distribution of conserved motifs in hARTC1. The blue region represents domain A, the red region represents the catalytic motif, and the purple region represents domain B. (**C**) Identities of the three regions between hARTCs and mARTCs. (**D**) Sequence alignment of hARTCs and mARTCs, and the results were displayed using ESPript. The R–S–EXE motif is marked in a black bold frame and residues that differ from this motif are highlighted in green.

A Neighbor-Joining (NJ) phylogenetic tree was constructed using MEGA6 (6140226) software to investigate the evolution of ARTC orthologs in different species. The tree consisted of six ARTC proteins from five species, including human, mouse, chicken, Xenopus, and zebrafish. ARTCs were divided into two major groups based on their sequence identity: ARTC1 and ARTC4 were in one branch; ARTC3, ARTC5, and ART2A/2B formed the other clade (Figure 1A). The ARTC family proteins are not highly conserved but differ significantly among species. The ARTC family proteins in the zebrafish have the strongest conservation (∼50% collinearity), and hARTC1 and mARTC1 have the closest genetic relationship (88% sequence similarity).

The crystal structure of rat ART2.2 has been determined. Based on the modeling structure from the Swiss-Model Repository (P52961), hARTC1 contains three regions: the N-terminal region, the catalytic domain, and the C-terminal region (Figure 1B). The identities of the three regions between hARTCs and mARTCs are 38%, 58%, and 18%, respectively (Figure 1C). The N-terminus and C-terminus of ARTCs are an α-helix-rich region and β-sheets, respectively, which are typical signal sequences for extracellular proteins and GPI-anchored membrane proteins. The catalytic domain contains a central six-stranded β-sheet and an α-helix that form the upper and lower parts of the active sites (Choe et al., 1992; Han et al., 1999; Mueller-Dieckmann et al., 2002). Sequence alignment of hARTCs and mARTCs showed that the conserved R–S–EXE motif is found in all the active ARTC enzymes. The most important Glu residue crucial for the catalytic activity is in the fifth β-strand, which is missing in hARTC3 and mARTC3, whereas Arg and Ser are positioned in the first and second β-strand, respectively. In hARTC4, mARTC4, and mARTC3, Arg in the first β-strand is substituted by other amino acids. Ser in the second β-strand of hARTC3 and mARTC3 is replaced by Leu. Another crucial Glu is positioned two residues upstream of the catalytic Glu of active ARTCs, which is missing in hARTC4 and is substituted by Asp in mARTC3 (Figure 1D). Thus, loss or substitution of key residues corresponding to the R–S–EXE motif causes ARTC3 and ARTC4 to lose their enzymatic activity.

### The expression, localization, and enzymology of the hARTC family

We examined the expression of ARTCs in 10 human tissue types, including the esophagus, liver, ovary, gallbladder, lung, thyroid, stomach, breast, testis, and skeletal muscle, by quantitative real-time polymerase chain reaction (qPCR) (Supplementary Figure S1A). Consistent with previous reports, *hARTC1* was most prominently expressed in skeletal muscles, and *hARTC3* and *hARTC5* were mostly expressed in the testis (Friedrich et al., 2006; Leutert et al., 2018). *hARTC4* showed a broader expression pattern with prominent signals in the testis. Collectively, the results revealed that hARTCs were expressed in a tissue-specific manner.

Next, we examined the localization of endogenous hARTCs in human cardiomyocyte cell line (AC16) with the indicated antibodies (generated by our lab, Supplementary Figure S1B) and an anti-calnexin antibody (ER marker protein) by immunofluorescent staining (Supplementary Figure S1C). Previous studies have reported that hARTC1 is mainly localized in the ER, where it mono-ADP-ribosylates GRP78/BiP (Fabrizio et al., 2015). Consistently, our results demonstrated that both hARTC1 and hARTC3 mostly co-localized with calnexin, distinct from hARTCs with known ecto-enzyme localization patterns. hARTC4 and hARTC5 also co-localized with calnexin to varying extents. Our findings showed that all hARTCs are localized to the ER to some extent, where active hARTC1 may be mainly involved in the MARylation of intracellular targets.

**Figure 2:**
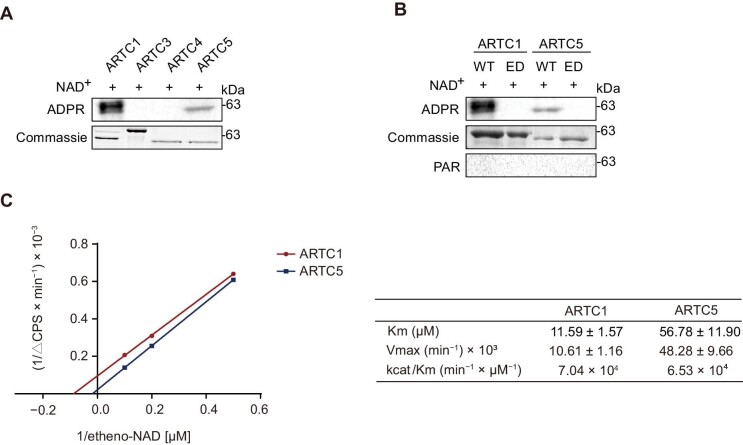
The analysis of hARTC auto-ADP-ribosylation. (**A**) Western blotting analysis of hARTC auto-ADP-ribosylation. The hARTCs were purified from *E. coli*, incubated with NAD^+^  *in vitro*, and then subjected to western blotting with an anti-poly/mono-ADP-ribose (anti-ADPR) antibody (*n* = 3 independent experiments). (**B**) Western blotting analysis of the auto-ADP-ribosylation of wild-type (WT) and enzymatically inactive (ED) hARTC1 and hARTC5 (*n* = 3 independent experiments). (**C**) Enzymatic kinetics for etheno-NAD^+^ of hARTC1 (red) or hARTC5 (blue). Recombinant hARTC1 or hARTC5 (10 μg/ml) was incubated at room temperature for 60 min at 37°C in the presence of 0, 2, 5, 10 μM etheno-NAD^+^, respectively. The ART activity was determined by measuring the increase in fluorescence intensity. Each data point represents mean ± SD from three replicates.

To analyze the enzymology of the hARTC family, we produced recombinant glutathione S-transferase (GST)-hARTC1 (N23–C295), GST-hARTC3 (N27–C362), GST-hARTC4 (N47–C285), and GST-hARTC5 (N23–C291) proteins in *Escherichia coli*. hARTCs were incubated with NAD^+^  *in vitro* to determine the ART activity. Using the anti-poly/mono-ADP ribose antibody, western blotting showed that hARTC1 and hARTC5 possessed auto-ADP-ribosylating activity, while hARTC3 and hARTC4 did not show such activity, consistent with the presence or absence of the R–S–EXE motif (Figure 2A). We constructed catalytically inactive hARTC1-E238/240G (hARTC1-ED) and hARTC5-E219/221G (hARTC5-ED) mutants, and the purified wild-type and mutant hARTC1 or hARTC5 from *E. coli* were used for the enzymatic activity assay *in vitro*. We found that both mutants lost the ART activity *in vitro* (Figure 2B). The mutants transfected into AC16 cells also did not show any auto-ADP-ribosylation (Supplementary Figure S1D). To further characterize hARTC1 and hARTC5, we examined their enzymatic kinetics using the etheno-NAD^+^ assay as previously described (Schultz et al., 2018). Recombinant proteins of hARTC1 and hARTC5 were incubated with etheno-NAD^+^  *in vitro* to measure the NAD^+^ hydrolase activity using etheno-NAD^+^(NAD^+^ analog) to produce a fluorescent product, which is examined in real-time by a fluorospectrophotometer. Km values for etheno-NAD^+^ of hARTC1 and hARTC5 were determined by measuring the increase in fluorescence intensity. Lineweaver-Burk analysis showed that the Km for etheno-NAD^+^ of recombinant hARTC1 and hARTC5 was 11.59 ± 1.57 μM (mean ± SD, *n* = 3) and 56.78 ± 11.90 μM (mean ± SD, *n* = 3), respectively (Figure 2C). Together, these results confirmed that hARTC1 and hARTC5 are catalytically active for auto-ADP-ribosylation.

### hARTC1 and hARTC3 form a heterodimer

**Figure 3:**
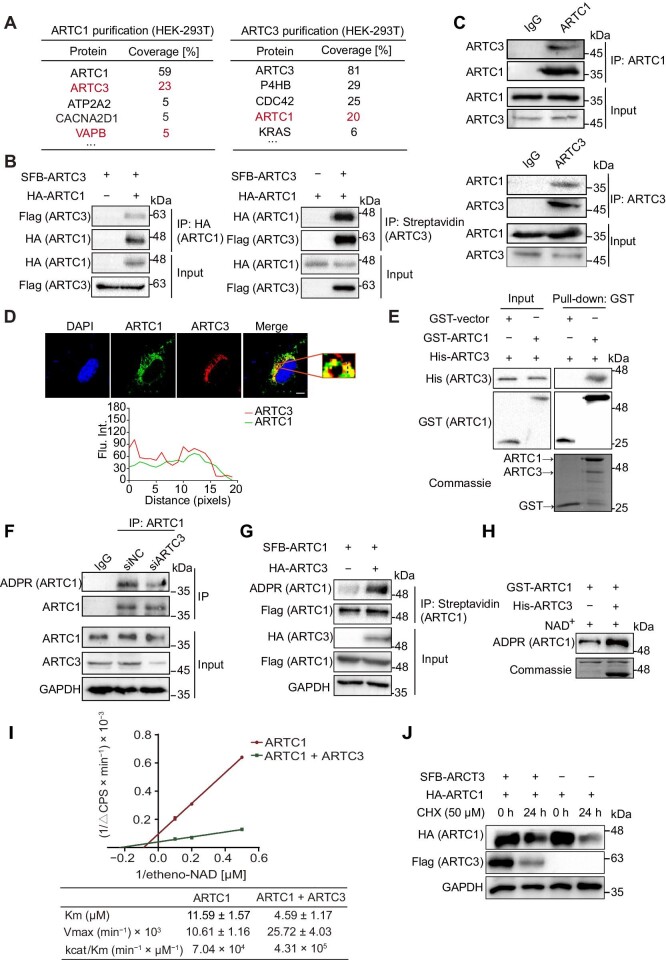
hARTC1 and hARTC3 form a heterodimer. (**A**) A list of hARTC1- or hARTC3-interacting proteins identified by MS. hARTC3 binds to hARTC1 reciprocally, as measured by the IP/MS assay. (**B**) Reciprocal co-IP analysis showed that hARTC1 interacts with hARTC3 in 293T cells (*n* = 3 independent experiments). (**C**) The interaction between endogenous hARTC1 and hARTC3 in AC16 cells was examined by co-IP assays (*n* = 3 independent experiments). (**D**) Representative immunofluorescence images of AC16 cells indicating that hARTC1 co-localizes with hARTC3. Shown in the red solid box is the zoomed-in image from the merged image. The percentage of hARTC1 and hARTC3 co-localizing with each other was quantified (*n* = 3 independent experiments). Scale bar, 5 μm. Flu. Int., fluorescence intensity. (**E**) hARTC1 interacts with hARTC3 *in vitro*. GST-hARTC1 or His-hARTC3 was purified from bacteria and incubated with each other. The bound proteins were analyzed by immunoblotting with the indicated antibodies. GST-hARTC1 and His-hARTC3 proteins were stained by Coomassie blue as loading controls (*n* = 3 independent experiments). (**F** and **G**) hARTC3 influences auto-ADP-ribosylation of hARTC1 *in vivo*. Lysates from AC16 cells were subjected to IP and immunoblotting analysis with the indicated antibodies. Knockdown of *hARTC3* impaired the auto-ADP-ribosylation of hARTC1 in AC16 cells (**F**), whereas hARTC3 overexpression enhanced the auto-ADP-ribosylation of hARTC1 *in vivo* (**G**). Shown are representative results from three independent experiments. (**H**) hARTC3 promotes the auto-ADP-ribosylation of hARTC1 *in vitro*. GST-hARTC1 and His-hARTC3 were incubated *in vitro*, and western blotting results showed that hARTC3 obviously promotes the auto-ADP-ribosylation of hARTC1 (*n* = 3 independent experiments). (**I**) Enzymatic kinetics for etheno-NAD^+^ of hARTC1 in the presence (green) or absence (red) of hARTC3 (*n* = 3 independent experiments). (**J**) Western blotting analysis showed the effect of hARTC3 on hARTC1 stability. AC16 cells were transfected with the indicated plasmids and treated with 50 μM CHX for the indicated time. GAPDH was used as the loading control (*n* = 3 independent experiments).

To gain mechanistic insights into the functions of hARTC1 in cells, we established cells stably expressing S-Flag-streptavidin binding protein (SFB)-tagged hARTC1 and performed tandem affinity purification and mass spectrometry (MS) analysis to identify its potential binding partners. The expression of hARTC1 was confirmed with western blotting and immunofluorescence staining (Supplementary Figure S2A and C). In the MS analysis, hARTC3 was found to be one of the most abundant proteins (Figure 3A, left table), indicating a potential interaction between hARTC3 and hARTC1. Conversely, abundant hARTC1 was also detected in the MS analysis of SFB-hARTC3 cells (Figure 3A, right table; Supplementary Figure S2B and C). To verify the results, we examined the interaction using exogenously tagged proteins. Reciprocal co-immunoprecipitation (co-IP) experiments confirmed the interaction between hARTC1 and hARTC3 (Figure 3B). Endogenous co-IP further confirmed the interaction between hARTC1 and hARTC3 in AC16 cells and 293T cells (Figure 3C; Supplementary Figure S2D). Immunofluorescent staining showed that hARTC1 co-localized with hARTC3 in AC16 cells (Figure 3D). We also purified GST-ARTC1 (N23–C295) and His-ARTC3 (N27–C362) from *E. coli* to perform pull-down assays and found that hARTC1 exhibited a direct interaction with hARTC3 (Figure 3E). We then characterized a complex of hARTC1 and hARTC3 using analytical size exclusion chromatography, confirming that hARTC1 and hARTC3 form a stable heterodimer (Supplementary Figure S2E). Further experiments are needed to determine how hARTC1 and hARTC3 interact with each other. These data strongly supported that hARTC3 is a binding partner of hARTC1.

To further investigate the functional impact of hARTC3 on hARTC1, we examined the auto-ADP-ribosylation of hARTC1 in AC16 cells with knockdown or overexpression of hARTC3. We found that knockdown of *hARTC3* significantly decreased the auto-ADP-ribosylation of hARTC1 (Figure 3F). Overexpression of hARTC3 could promote the auto-ADP-ribosylation of hARTC1 (Figure 3G), which was also found in the *in vitro* assay (Figure 3H), suggesting that hARTC3 cooperates with hARTC1 and contributes to activating the enzymatic activity of hARTC1 (Figure 3I). We then investigated whether hARTC3 enhanced the enzymatic activity of hARTC1 by stabilizing it. We treated cells with cycloheximide (CHX) to inhibit new protein synthesis and measured the rate of hARTC1 degradation at the indicated time points. Our results revealed that ectopic expression of hARTC3 led to a decrease in hARTC1 degradation (Figure 3J), indicating that hARTC3 may inhibit hARTC1 degradation and stabilize hARTC1.

Arginine-specific ADP-ribosylation is a dynamic and reversible post-translational modification that is mediated by ARTC1 and hydrolyzed specifically by ADP-ribosylarginine hydrolase 1 (ARH1) (Rack et al., 2018). To confirm the auto-ADP-ribosylating activity of hARTC1, we disrupted the expression of endogenous ARH1 with siRNA targeting *ARH1* in 293T and AC16 cells and confirmed the loss of endogenous ARH1 by western blotting (Supplementary Figure S3A and B). We found that disrupting the de-MARylation by knockdown of *ARH1* remarkably increased the MARylation of hARTC1, suggesting that hARTC1 is auto-ADP-ribosylated and the MARylation of hARTC1 is arginine-specific (Supplementary Figure S3C and D).

### hARTC1 regulates calcium homeostasis and ADP-ribosylates VAPB

**Figure 4:**
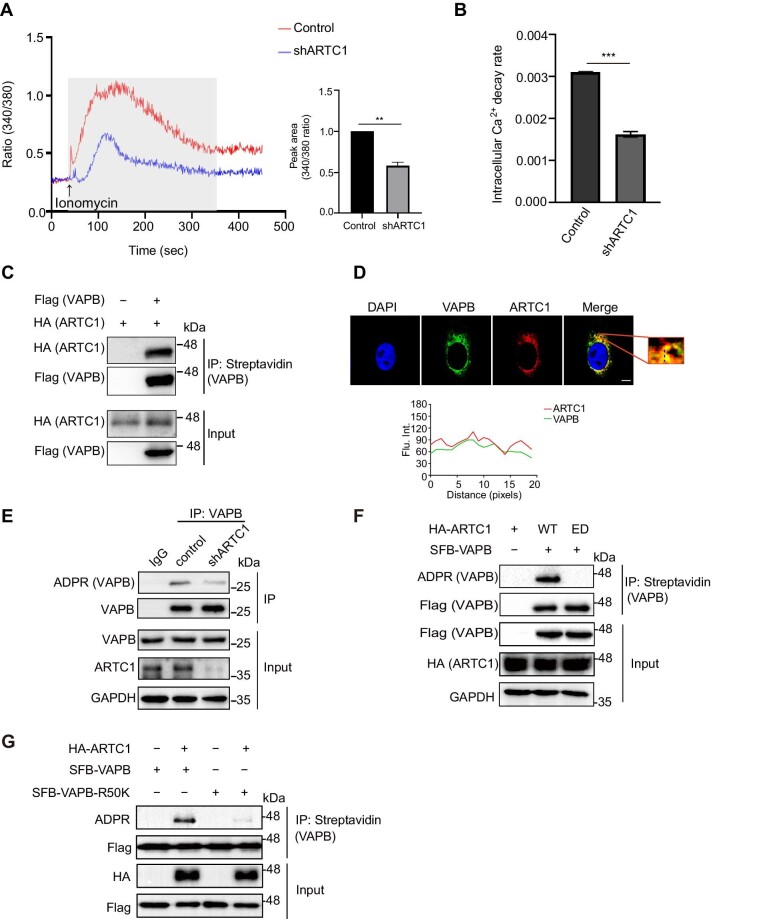
hARTC1 regulates calcium homeostasis and ADP-ribosylates VAPB. (**A** and **B**) Ionomycin (1 μM)-triggered Ca^2+^ signaling in wild-type (Control) and *hARTC1*-knockdown (shARTC1) cells. Fura-2 (340/380 ratio) was used to indicate the intracellular Ca^2+^ concentration. Histograms show the average peak area (**A**) and intracellular Ca^2+^ decay rate (**B**) with ionomycin-triggered Ca^2+^ transients in each group (*n* = 3 independent experiments). **P < 0.01; ***P < 0.001. (**C**) hARTC1 interacts with human VAPB in AC16 cells. Lysates from AC16 cells transfected with the indicated plasmids were subjected to IP and immunoblotting analysis with the indicated antibodies (*n* = 3 independent experiments). (**D**) VAPB co-localizes with hARTC1 in AC16 cells. Immunofluorescence staining was performed using antibodies against hARTC1, hARTC3, or VAPB. Shown in the red solid box is the zoomed-in image from the merged image. The percentage of hARTC1 and VAPB co-localizing with each other was quantified (*n* = 3 independent experiments). Scale bar, 5 μm. (**E**) Knockdown of *hARTC1* reduces the ADP-ribosylation of VAPB. IP and immunoblotting were performed with the indicated antibodies and showed the VAPB ADP-ribosylation levels in *hARTC1*-knockdown and control AC16 cells (*n* = 3 independent experiments). (**F**) Enzymatically inactive (ED) hARTC1 fails to ADP-ribosylate VAPB (*n* = 3 independent experiments). (**G**) Identification of Arg50 of VAPB as a key ADP-ribosylation site using wild-type VAPB and the R50K mutant (*n* = 3 independent experiments).

Previous reports showed that *mARTC1*-knockout mice were viable and developed normally but displayed reduced skeletal muscle ADP-ribosylation and signs of muscle weakness. Therefore, we aimed to investigate the function of hARTC1 in heart muscle cells. We established stable *hARTC1*-knockdown AC16 cells (Supplementary Figure S4A) and then performed intracellular Ca^2+^ property assays with a dual-excitation fluorescence photomultiplier system. We observed a significant decrease in intracellular Ca^2+^ concentration as well as intracellular Ca^2+^ decay rate upon knockdown of *hARTC1* (Figure 4A and B), suggesting that the lack of hARTC1 might affect heart muscle function and hinting a potential role for hARTC1-mediated ADP-ribosylation in heart muscles. We also examined the effect of hARTC3 on hARTC1-mediated intracellular calcium homeostasis.

We ectopically co-expressed hARTC3 and hARTC1 in AC16 cells (Supplementary Figure S4B) and observed a more significant increase in intracellular Ca^2+^ concentration and Ca^2+^ decay rate compared to AC16 cells overexpressing hARTC1 alone (Supplementary Figure S4C and D). Notably, hARTC3 alone caused a slight increase in Ca^2+^ concentration. Furthermore, we are currently conducting further investigations into the biological function of hARTC3, which is beyond the scope of this study.

Next, we investigated the ADP-ribosylated proteins mediated by hARTC1. Based on our previous IP/MS results (Figure 3A), we identified VAPB as another protein of interest. VAPB is a member of the human VAP family, which is involved in cellular calcium homeostasis regulation (De Vos et al., 2012; Lindhout et al., 2019). Exogenous co-IP assays demonstrated that hARTC1 interacted with VABP in AC16 cells (Figure 4C). Immunofluorescence staining also showed that hARTC1 co-localized with VAPB (Figure 4D). The ADP-ribosylation of VAPB was dramatically reduced in *hARTC1*-knockdown cells compared to the control group (Figure 4E), suggesting VAPB as a direct target of hARTC1. Next, we analyzed the ADP-ribosylation of VAPB in *hARTC1*-knockdown AC16 cells reconstituted with wild-type hARTC1 or catalytically inactive hARTC1-ED (E238/240G) mutant (Figure 4F). We found that wild-type hARTC1 could ADP-ribosylate VAPB, while the hARTC1-E238/240G mutant could not, suggesting that hARTC1 mediates the ADP-ribosylation of VAPB in cardiac muscle cells.

We also identified the ADP-ribosylation sites of VAPB. Previous studies identified ADP-ribosylation on Arg50 and Arg197 of mouse VAPB (Leutert et al., 2018). Based on homology alignment (Supplementary Figure S5A), we predicted Arg50 and Arg197 of human VAPB as potential ADP-ribosylation sites and generated the R50K, R197K, and R50KR197K double mutants. The ADP-ribosylation of R50K was markedly abolished, while that of R197K was not affected (Figure 4G; Supplementary Figure S5B), indicating that Arg50 is the primary modification site of human VAPB. Collectively, our results demonstrated that VAPB is a target of hARTC1 and Arg50 is the major ADP-ribosylation site.

### ADP-ribosylated VAPB is involved in calcium homeostasis and VAPB–SCRN1 interaction

**Figure 5:**
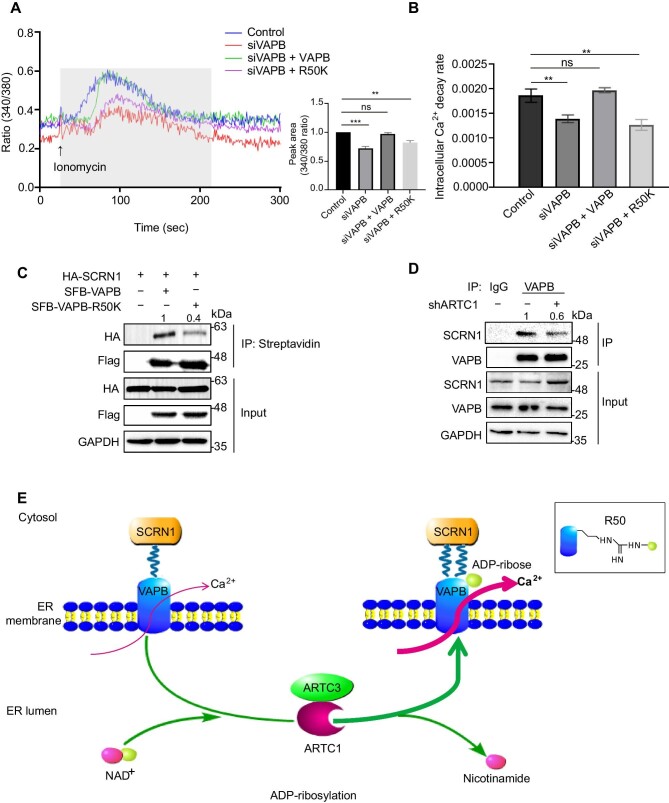
ADP-ribosylated VAPB is involved in calcium homeostasis and the VAPB–SCRN1 interaction. (**A** and **B**) Ionomycin (1 μM)-triggered Ca^2+^ transients in control cells, *VAPB*-knockdown cells, and *VAPB*-knockdown cells reconstituted with wild-type VAPB or the R50K mutant. Histograms display the average peak area (**A**) and intracellular Ca^2+^ decay rate (**B**) with ionomycin-triggered Ca^2+^ transients in each group. Data shown are representative of at least three independent experiments. **P < 0.01; ***P < 0.001; ns, no significance. (**C**) The ADP-ribosylation of VAPB is important for its interaction with SCRN1. Lysates from AC16 cells transfected with SCRN1 and wild-type VAPB or the VAPB-R50K mutant were subjected to IP and immunoblotting analysis with the indicated antibodies (*n* = 3 independent experiments). (**D**) The endogenous interaction of VAPB with SCRN1 is decreased in *ARTC1*-knockdown cells. Lysates from AC16 cells were subjected to IP and immunoblotting analysis with the indicated antibodies (*n* = 3 independent experiments). (**E**) A working model depicting that hARTC1 ADP-ribosylates VAPB and regulates calcium homeostasis.

ER is the main intracellular Ca^2+^ store and plays a central role in intracellular Ca^2+^ mobilization and dynamics (Berridge et al., 2003). VAPB is an integral ER membrane protein that regulates Ca^2+^ exchange between the ER and other intracellular membranes. We aimed to examine whether the ADP-ribosylation of VAPB, mediated by hARTC1, regulated calcium homeostasis. Firstly, we knocked down *hVAPB* in AC16 cells (Supplementary Figure S6A) and reconstituted the cells with wild-type VAPB or the R50K mutant. After confirming the comparable expression levels of wild-type and mutant VAPB by western blotting (Supplementary Figure S6B), we examined intracellular Ca^2+^ dynamics in these cells. The results indicated that knockdown of *hVAPB* led to a significant decrease in intracellular Ca^2+^ level and Ca^2+^ decay rate. Moreover, the alterations could be restored to the control level after complementing wild-type VAPB, but not the R50K mutant (Figure 5A and B). To further investigate the importance of Arg50 ADP-ribosylation of VAPB in regulating calcium homeostasis, we used the CRISPR–Cas9 system to knock out endogenous *VAPB* in AC16 cells and confirmed the loss of endogenous VAPB by western blotting (Supplementary Figure S6C). Next, we reconstituted VAPB-knockout (VAPB-KO) cells with wild-type VAPB or the R50K mutant and examined the exogenous VAPB expression levels by western blotting (Supplementary Figure S6D). These cells were then treated with the ARTC inhibitor novobiocin or mock-treated. Upon ARTC1 inhibition, the intracellular Ca^2+^ level and Ca^2+^ decay rate in VAPB-KO cells reconstituted with wild-type VAPB were significantly decreased, similar to that in the R50K-reconstituted cells (Supplementary Figure S6E and F), suggesting that VAPB-regulated intracellular Ca^2+^ flux occurs in an ADP-ribosylation-dependent manner.

Previous studies have shown that VAPB can regulate intracellular calcium homeostasis by interacting with the cytoplasmic protein SCRN1 (Lindhout et al., 2019). We performed interactive modeling with the SCRN1 ‘FFAT’ motif and MSP domain of VAPB (PDB 3IKK) using the software Pymol. As shown in Supplementary Figure S7, ADP-ribosylated Arg50 is highlighted with a blue stick, and the SCRN1 ‘FFAT’ motif (401–407 aa) is highlighted with a red stick. When Arg50 of VAPB is ADP-ribosylated by hARTC1, the adenosine ring of ADPr and the aromatic ring of Tyr403 form π–π interaction, which might enhance the interaction of VAPB and SCRN1. To test this possibility, we performed exogenous co-IP assays to measure the interactions between wild-type VAPB or VAPB- R50K mutant and SCRN1. The result indicated that ADP-ribosylation-defective VAPB strongly decreased its SCRN1-binding capacity as compared to wild-type VAPB (Figure 5C). Next, we examined the endogenous interaction between VAPB and SCRN1 in *ARTC1*-knockdown or control cells and obtained the similar result (Figure 5D), suggesting that the ADP-ribosylation of VABP might regulate its SCRN1-binding capacity. Thus, ADP-ribosylated VAPB by ARTC1 promotes its interaction with SCRN1 and modulates intracellular calcium homeostasis (Figure 5E).

## Discussion

In this study, we systematically investigated the hARTC family. The presence of respective orthologs in many mammalian species suggests that these genes were generated by duplication events at or before the mammalian radiation. Most hARTCs exhibit a tight tissue-specific expression pattern, which may reflect their regulatory function in specific tissues. We further unveiled the ER localization of hARTCs and thus speculated on their potential functions in the ER. Enzymology assays with recombinant hARTCs confirmed the arginine-specific transferase activity for hARTC1 and hARTC5, while hARTC3 and hARTC4 lacking the R–S–EXE motif failed to display any detectable arginine-specific enzyme activity.

We focused our research on hARTC1 and found that hARTC1 could form a heterodimer with hARTC3. Notably, hARTC3 enhanced the enzymatic activity of hARTC1 through stabilizing it, thereby regulating hARTC1-mediated intracellular calcium homeostasis in human heart muscle cells. Leutert et al. (2018) reported that mARTC1-deficient mice showed muscle weakness characterized by a reduced resistance of the muscle activity, suggesting that the lack of mARTC1 affects skeletal or heart muscle function in mice. Consistently, our results indicated that hARTC1 might play a key role in heart muscle cells by regulating intracellular calcium homeostasis. Additionally, hARTC1-mediated calcium homeostasis was promoted by hARTC3. The biological function of inactive hARTC3 has remained unknown, so our work not only confirmed the role of hARTC1 in regulating intracellular calcium homeostasis but also identified a novel function of hARTC3 in stabilizing hARTC1.

The profiling work of mARTC1-mediated ADP-ribosylomes in mice revealed a large basal ADP-ribosylome that consisted of 558 ADP-ribosylated proteins, which were constrained to the extracellular compartment, plasma membrane, ER, and mitochondria with modification of many low-abundant proteins (Leutert et al., 2018). However, hARTC1-mediated ADP-ribosylomes in humans have not been extensively investigated, and only a few targets of hARTC1 have been identified so far. For example, hARTC1 mono-ADP-ribosylates HNP-1 on Arg14 and Arg24, altering its biological activity (Paone et al., 2002). Another substrate of hARTC1 is the basic fibroblast growth factor 2 (FGF-2), which is located on the cell surface and in the extracellular matrix. The ADP-ribosylation of FGF-2 in its receptor-binding domain may inhibit its binding to the receptor (Jones and Baird, 1997). Additionally, hARTC1 contributes to the MARylation of platelet-derived growth factor-BB (PDGF-BB), impairing its binding capacity to PDGF receptors and abolishing its ability to stimulate mitogenic and chemotactic responses in human pulmonary smooth muscle cells (Saxty et al., 2001). Lastly, hARTC1 contributes to GRP78/BiP MARylation, inhibiting the translation during ER stress response (Fabrizio et al., 2015). Recently, Hesse et al. (2022) identified human CD73 as a target for ARTC1-mediated MARylation. Our work adds to the limited pool of hARTC1 substrates.

We believe that there are additional targets of hARTC1 on the ER, based on the following evidence. Firstly, the majority of the identified mARTC1-mediated intracellular arginine-specific ADP-ribosylation is detected in the endomembrane system, likely corresponding to protein modification occurring in the ER and Golgi (Leutert et al., 2018). Secondly, hARTC1 is localized in the ER. Indeed, we identified a new ADP-ribosylated ER-resident substrate, VAPB, by hARTC1. Although mouse VAPB is known to be ADP-ribosylated at Arg50 and Arg197, arginine-specific ADP-ribosylation of human VAPB has not been reported, nor has the role of the modification of VAPB been characterized. VAPB is a member of the two ER-resident tail-anchored VAP families (VAPA and VAPB) that are generally involved in forming ER contacts with other membranes by directly interacting with the FFAT motif in target-tethering factors (Lev, 2010; Helle et al., 2013; Phillips and Voeltz, 2016). VAPB directly interacts with FIP200 and ULK1 via their conserved FFAT motifs to modulate autophagosome biogenesis (Zhao et al., 2018). VAPB also binds to an outer mitochondrial membrane protein, PTPIP51, and promotes IP3R/VDAC1-mediated ER/mitochondrion contacts for Ca^2+^ delivery (De Vos et al., 2012; Gomez-Suaga et al., 2017).

Recently, Lindhout et al. (2019) reported that the interaction between SCRN1 and VAPB was involved in modulating the VAP-associated phenotypes, such as ER continuity, ER dynamics, and calcium homeostasis. It has also been shown that P56S and T46I mutations of VAPB cause a familial ALS by eliminating the native MSP structure, leading to insoluble cytosolic aggregation and loss of functions under physiological conditions. We found that the ADP-ribosylated site of human VAPB was Arg50, which was close to Pro56 and Thr46. Thus, we speculated that the lack of Arg50 ADP-ribosylation of VAPB might lead to insoluble cytosolic aggregates. However, we did not observe the formation of insoluble aggregates in the VAPB-R50K mutant (data not shown). Here, we discovered that the ADP-ribosylation of VAPB facilitated its interaction with SCRN1, suggesting that the excitatory function of ARTC1-mediated ADP-ribosylation of VAPB could be crucial in regulating calcium homeostasis by controlling the binding of VAPB to SCRN1. Conversely, the absence of ADP-ribosylation of VAPB hindered its interaction with SCRN1, leading to disruptions in intracellular calcium homeostasis and decreased clearance in intracellular Ca^2+^. Such disruptions are likely to be particularly damaging to the muscle contractile function, which is heavily dependent on Ca^2+^ signaling. In addition, we identified another substrate of ARTC1, calcium voltage-gated channel auxiliary subunit α2δ 1 (CACNA2D1), which interacts with ARTC1 and can be mono-ADP-ribosylated. Unfortunately, we have not identified the ADP-ribosylation sites of CACNA2D1. Further investigations are needed.

In summary, we propose that hARTC1 plays a significant role in regulating calcium homeostasis in heart muscle cells. hARTC1-mediated ADP-ribosylation of VAPB affects its interaction with SCRN1 and consequently regulates calcium homeostasis, which may act as a novel mechanism for hARTC1-mediated ADP-ribosylation in heart muscle cells. Future work is required to better understand the molecular function of hARTC1–hARTC3 interaction in mediating Ca^2+^-driven muscle contractile function. Finally, investigating additional targets of hARTC1 that are involved in modulating the muscle contractile function is required to gain more insights into the precise functional implications of hARTC1-mediated MARylation in muscle tissues.

## Materials and methods

### Cell culture

Human embryonic kidney 293T (HEK-293T) (ATCC®, CRL-11268™) cell line and human cardiomyocyte cell line AC16 (Sigma, SCC109) were obtained from the Cell Bank of Type Culture Collection of Chinese Academy of Sciences (Shanghai). The cell lines were confirmed to be free of mycoplasma contamination. Cells were cultured in Dulbecco's modified Eagle's medium (GIBCO) supplemented with 10% fetal bovine serum (GEMINI, 900-108), 100 μg/ml penicillin, and 100 μg/ml streptomycin at 37°C with 5% CO_2_ in a humidified incubator.

### Tissue preparation

A total of 10 normal human tissue samples used in the study were provided by the Affiliated Hospital of Hebei University (Baoding, China). All subjects gave their informed consent for inclusion before they participated in the study. All participants were scheduled for a face-to-face interview after written informed consents were obtained. This study was conducted in accordance with the Declaration of Helsinki, and the protocol was approved by Institutional Review Boards of Hebei University (Ethics Committee of Hebei University: HDFY-LL-2022-020). The relevant clinical information of the samples is shown in Supplementary Table S1.

### Phylogenetic analysis

Phylogenetic tree of ARTC proteins was generated with sequences from human, mouse, chicken, Xenopus, and zebrafish using the NJ method by MEGA6 (6140226). The secondary structure of protein was predicted by the website http://www.sbg.bio.ic.ac.uk/phyre2/html/page.cgi?id=index. Sequence alignment was generated via ClustalX1.83 using default parameters, and the results were displayed using ESPript (http://espript.ibcp.fr/ESPript/cgi-bin/ESPript.cgi).

### Plasmid construction and protein purification

hARTC1 and hARTC3 were cloned into the SFB vector (SFB tag; see Supplementary Figure S8 for detailed vector construction methods) or phage-HA vector (HA tag), respectively. hARTC1-N23–C295, hARTC3-N27–C362, hARTC4-N47–C285, and hARTC5-N23–C291 were cloned into the pGEX-4T-1 vector with an N-terminal GST for purification of recombinant proteins GST-ARTC1 (N23–C295), GST-ARTC3 (N27–C362), GST-ARTC4 (N47–C285), and GST-ARTC5 (N23–C291). hARTC3-N27–C362 was also cloned into the pET-15b vector with an N-terminal His tag. Human full-length VAPB and the R50K mutant were cloned into the SFB vector. Human full-length SCRN1 was cloned into the phage-HA vector.

Recombinant proteins were expressed as GST or His fusions in the respective vectors in *E. coli* BL21 (Codon Plus) at 37°C. Protein expression was induced by adding 0.12 mM IPTG at 22°C for 16 h. Subsequently, the cells were lysed by sonication in ice-cold lysis buffer (25 mM Tris–HCl, pH 8.0, 400 mM NaCl, and 5% glycerol). After centrifugation, the supernatant containing recombinant proteins were loaded onto Glutathione Sepharose 4B beads (GE Healthcare, 17-5132-02) or Ni-chelating Sepharose (GE Healthcare, 17-0575-02) pre-equilibrated with lysis buffer. All bound proteins were eluted using elution buffer (25 mM Tris–HCl, pH 8.0, 400 mM NaCl, 5% glycerol, and 250 mM imidazole).

### Antibodies

The following antibodies were purchased: anti-Flag (MBL, M185-3L), anti-HA (MBL, M180-3), anti-VAPB (Proteintech, 14477-1-AP and 66191-1-Ig), anti-SCRN1 (Proteintech, 14303-1-AP), anti-GAPDH (Proteintech, 60004-1-Ig and 10494-1-AP), anti-poly/mono-ADP-ribose (Cell Signaling Technology, 83732S), PAR/pADPr (R&D Systems, 4335-MC-100), anti-Calnexin (Novus, NB300-518), anti-His (Proteintech, 66005-1-Ig), anti-GST (Proteintech, 66001-2-Ig), DyLight 488-conjugated goat anti-mouse IgG (Abbkine, A23210), Dylight 594-conjugated goat anti-rabbit IgG (Abbkine, A23420), horseradish peroxidase (HRP)-conjugated mouse anti-rabbit IgG LCS (Abbkine, A25022), HRP-conjugated goat anti-mouse IgG (Proteintech, 15014), and HRP-conjugated goat anti-rabbit IgG (Proteintech, 15015).

Antibodies against ARTC1, ARTC3, ARTC4, and ARTC5 were generated by immunizing rabbits with recombinant proteins GST-ARTC1 (N23–C295), GST-ARTC3 (N27–C362), GST-ARTC4 (N47–C285), and GST-ARTC5 (N23–C291), respectively, purified in our laboratory. Serum samples were obtained from immunized rabbits and incubated with AminoLink Plus Coupling Resin (Thermo, 20501) in Pierce Disposable Plastic Columns (Thermo, 29922). The resin was washed three times with PBS. Antibodies were eluted by 100 mM Glycine (pH 2.5).

### Generation of stable cell lines

AC16 cells were transduced with lentiviral vector carrying an inducible short hairpin RNA (shRNA) to hARTC1 for reduced expression. Cells with stable integration of shRNA constructs were selected with puromycin (1 μg/ml) for ∼1 week. The shRNA targeted sequence for hARTC1 was 5′-GCGAGTACATCAAAGACAAGA-3′.

293T cells were transfected with SFB-tagged *hARTC1* or *hARTC3* expression construct, cultured in medium containing 1 μg/ml puromycin for 7 days, and selected for stably expressing cells. SFB-hARTC1 and SFB-hARTC3 expression levels were confirmed by western blotting and immunofluorescence.

CRISPR sgRNA sequence (5′-ACAGCGGAATCATCGATGCA-3′) targeting exon 2 of *VAPB* gene was cloned into lentiCRISPR v2 plasmid. CRISPR plasmid combined with two packaging plasmids, psPAX2 and pMD2.G, were transfected into HEK-293T cells to produce the virus. Supernatants containing viruses were collected 48 h after transfection and infected AC16 cells in the presence of 10 μg/ml polybrene. After infecting for 6 h, supernatants were substituted with fresh culture medium. Two days later, the infected AC16 cells were selected with 1 μg/ml puromycin. Then, the VAPB-KO stable cell line was screened by the single clonal method, and the knockout efficiency was tested using western blotting.

### RNA interference

The siRNA sequences targeting *ARTC3, ARTC4, ARTC5, VAPB*, and *ARH1* were as follows: CGUGUUGGCCAAAGUCACUTT (*ARTC3*), GGCUGUAGCAAACAGGUUATT (*ARTC4*), CCUUGUACUGGGAGUUGAATT (*ARTC5*), CCACCAUAUCAUGGGAUUUTT (*VAPB*), and GGGUACUACAAUGGGAAGUTT (*ARH1*). siRNAs were transfected into cells using Lipofectamine 2000 (Thermo, 11668019) according to the manufacturer's instructions.

### Protein affinity purification and MS analysis

To find hARTC1- and hARTC3-binding partners, we harvested HEK-293T cells stably expressing SFB-hARTC1 and SFB-hARTC3 and washed cells with phosphate-buffered saline (PBS). Cells were lysed with 30 ml of ice-cold NETN 100 buffer (100 mM NaCl, 20 mM Tris–HCl, pH 8.0, 0.5 mM EDTA-2Na, 0.5% NP-40). The pellets were incubated with NETN 300 buffer (300 mM NaCl, 20 mM Tris–HCl, pH 8.0, 0.5 mM EDTA-2Na, 0.5% NP-40). The soluble fraction was incubated with 0.5 ml High Capacity Streptavidin Agarose (Thermo, 20359) beads for 4 h. The beads were washed three times with NETN 100 buffer. Associated proteins were eluted with 2 mM Biotin (Sigma, B4501) in PBS and further incubated with 0.05 ml S beads (MERCK, 69704). The bound proteins were eluted with sodium dodecyl sulfate (SDS) loading buffer and subjected to 10% SDS–polyacrylamide gel electrophoresis (PAGE). The entire protein band (<1 cm) was excised and analyzed by MS.

### qPCR

Total RNA was extracted by Trizol reagent (Sigma, T9424) and transformed into cDNA by the reverse transcription kit (Invitrogen, 18080051). qPCR analysis was repeated three times on Bio-Rad CFX96 using SYBR premix ex Taq (TaKaRa, RR820). All qPCR data were standardized by *GAPDH*. The primer information is shown in Supplementary Table S2.

### Co-IP and western blotting assay

All cells were briefly rinsed with ice-cold PBS before collection. Cells were lysed in NETN 300 buffer. The lysates were cleared by centrifugation (13000 rpm for 10 min at 4°C) and subjected to IP with Protein G-Sepharose beads (Thermo, 101243) with 1 μg mouse anti-HA or with High Capacity Streptavidin Agarose for overnight. The beads were centrifuged and washed three times with NETN 100 buffer. Bound proteins were eluted by boiling, resolved by 10% SDS–PAGE (unless otherwise indicated), and transferred to PVDF membranes (Merck Millipore). For western blotting, the membranes were blocked with 5% non-fat dry milk and then incubated with the indicated antibodies, followed by incubation with HRP-labelled secondary antibodies and detection using the ECL western blotting detection reagents (Thermo, 34577). All western blotting experiments were performed according to standard procedures.

### Immunofluorescence staining

Cells were grown on coverslips in 6-well plates and transfected with the indicated plasmids or siRNA using Lipofectamine 2000. The cells were fixed in 4% paraformaldehyde (Sigma, P6148) and then permeabilized in 1% Triton X-100 (Sigma, X100) for 5 min. After washing with PBS, cells were incubated with the indicated primary antibodies for 1 h at room temperature. After extensive washes with PBS, cells were incubated with Alexa Fluor 488- or Alexa Fluor 594-conjugated anti-mouse IgG or anti-rabbit IgG antibodies for 30 min at room temperature. After PBS wash, the nuclei were stained with DAPI (Sigma, D9542; 1:10000 in PBS). The coverslips were mounted onto glass slides and visualized with a confocal laser scanning microscope (OLYMPUS, FV3000).

The co-localization analysis of hARTC enzymes with calnexin or other proteins in the cells was performed with cellSens V3.2 software (OLYMPUS). The cellSens V3.2 was used to select positive regions as regions of interest, and fluorescence intensity was calculated by ImageJ.

### ADP-ribosylation assay

Cells lysates were used for IP with High Capacity Streptavidin Agarose for 4 h. After washing three times with NETN 100 buffer, the samples were boiled in SDS loading buffer and analyzed by western blotting using an anti-poly/mono-ADP-ribose antibody.

For the purified recombinant proteins, the assay was performed in a total of 50 μl reaction mixture containing 20 mM Tris–HCl (pH 8.0), 5 mM DTT, 10 μM NAD^+^ (Sigma), and recombinant hARTCs (1.7 μM) for 60 min at 37°C. After mixed with SDS loading buffer and boiled at 100°C for 10 min, the samples were run on an SDS–PAGE gel, stained with Coomassie blue, and subjected to western blotting analysis.

### Determination of enzymatic kinetic values

For determination of enzymatic kinetics, recombinant hARTCs were incubated with etheno-NAD^+^ (0, 2, 5, 10 μM) (Sigma, N2630) for 60 min at 37°C in 20 mM Tris–HCl (pH 8.0). Fluorescence intensity was measured by SpectraMax i3x (Molecular Devices). The excitation wavelength was 310 nm, and the emission was recorded at 403 nm. The fluorescence intensity was expressed as photons per second (CPS).

### GST pull-down assay

GST or GST-hARTC1 (N23–C295) and His-hARTC3 (N27–C362) were incubated with Glutathione Sepharose 4B beads (GE Healthcare, 17-5132-02) and Ni-chelating Sepharose (GE Healthcare, 17-0575-02), respectively, at 4°C for 2 h with rotation. After washing three times with NETN 100 buffer, the samples were boiled in SDS loading buffer and analyzed by 10% SDS–PAGE followed by western blotting using anti-His and anti-GST antibodies.

### Size exclusion chromatogrphy

Purified GST-ARTC1 (N23–C295) and His-ARTC3 (N27–C362) were incubated at a molar ratio of 1:3 at 37°C for 1 h. Then, the mixture or the indicated protein was analyzed separately through the HiLoad™ 16/600 Superdex™ 75 pg column (GE Healthcare, 28-9893-33), equilibrated with the buffer containing 25 mM Hepes (pH 7.0), 150 mM NaCl, and 5% (*v*/*v*) glycerol, at a flow rate of 1.0 ml/min. The elutions were collected and analyzed by 10% SDS–PAGE.

### Intracellular Ca^2+^ transient analysis

Intracellular Ca^2+^ concentration was measured with the intracellular Ca^2+^ indicator Fura-2/AM (Sigma, 47989). AC16 cells were loaded with Fura-2/AM (2 μM) for 20 min at 37°C, and fluorescence intensity was recorded with a dual-excitation fluorescence photomultiplier system (IonOptix). Cells were placed onto an Olympus IX-70 inverted microscope and imaged through a Fluor 40× oil objective. Cells were exposed to light excited by a 75-W lamp and passed through either a 340-nm or a 380-nm filter. Fluorescence emissions were detected between 480 nm and 520 nm, and the qualitative change in Fura-2 fluorescence intensity (FFI) was inferred from the FFI ratio at the two wavelengths (340 nm/380 nm). Fluorescence decay rate was calculated as an indicator of intracellular Ca^2+^ clearance (Guo et al., 2013). Ca^2+^ release from ER stores was triggered by application of 1 μM ionomycin (Sigma, I3909).

### Data analysis

Immunofluorescence images were obtained by the OLYMPUS FV3000 confocal laser scanning microscope (60XO, NA 1.4). The experiments were repeated at least three times. The values were expressed as mean ± standard deviation (SD). The differences among the groups were analyzed by *t*-test with GraphPad Prism or SPSS 19.0 software. *P* ≤ 0.05 was considered significant unless otherwise indicated.

The crystal structure of VAPB (1–125 aa, PDB 3IKK) was obtained from PDB, and the structure of SCRN1 (401–407 aa) was obtained by modeling. The complex structures of SCRN1 and VAPB were visualized and annotated using PyMOL v1.7.2.1.

## Supplementary Material

mjad043_Supplemental_File

## Data Availability

The additional data that support the findings of this study are available from the corresponding author on request.

## Notes

**Author contributions:** C.W., X. Yu, and Z.Y. designed the experiments. X.M., M.L., Y. Liu, X.Z., Y.W., Y. Li, and J.W. performed the experiments. C.W., Z.Y., X.M., Y. Liu, and X.Z. analyzed the data. X.L. and X. Yang made structural modeling. C.W. wrote the manuscript. All authors read and approved the final manuscript.
