# Supplementary material for: ARTC1-mediated VAPB ADP-ribosylation regulates calcium homeostasis
Source: J Mol Cell Biol. 2023 Jun 28;15(7):mjad043. doi: 10.1093/jmcb/mjad043 (PMC10928986; doi:10.1093/jmcb/mjad043)
Supplement: mjad043_Supplemental_File [file mjad043_supplemental_file.pdf]

Supplementary Figures

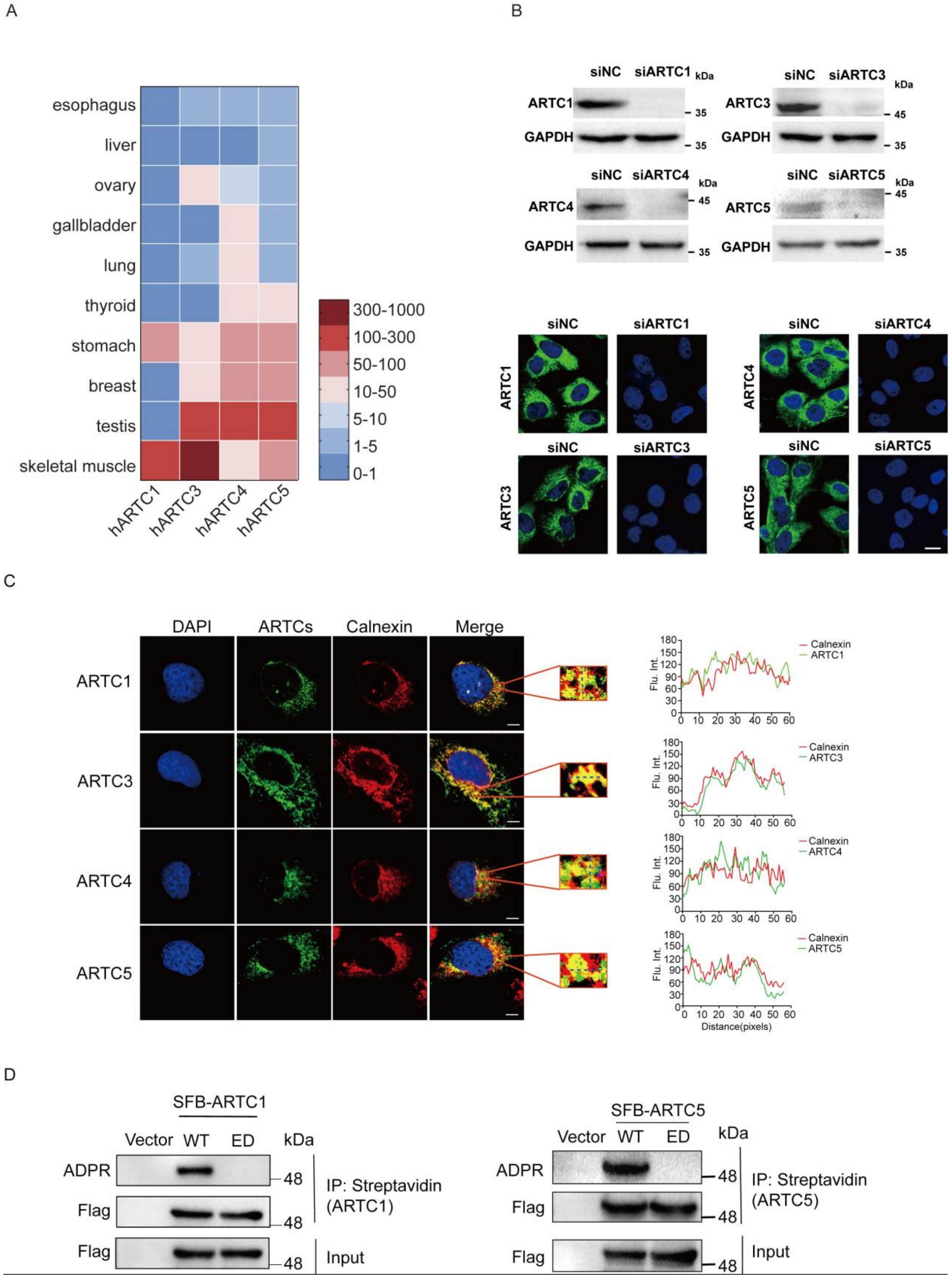

Supplementary Figure S1 Expressions, localizations and enzymatic activities of hARTCs. (**A**) mRNA

levels of *hARTCs* in different tissues were measured by quantitative real-time PCR (n = 3 independent experiments). (**B**) The specificity of generated ARTCs antibodies was tested by western blotting and immunofluorescent staining against hARTC1, 3, 4, 5. AC16 cells were transfected with empty vectors or siARTCs as indicated, and then subjected to western blotting or immunofluorescent staining. Scale bar = 10 μm. (**C**) Representative immunofluorescence images of AC16 cells stained with anti-calnexin (Red) or anti-ARTC (Green) antibodies. Nuclear counterstaining was carried out using DAPI. The zoom-in images in the red solid boxes show the co-localization of the different ARTCs and calnexin, which was quantified (n = 3 independent experiments). Scale bar = 5 μm. Flu. Int.: Fluorescence Intensity. (**D**) Auto-ADP-ribosylations of the wild-type hARTC1/5 and their mutants in AC16 cells were examined.

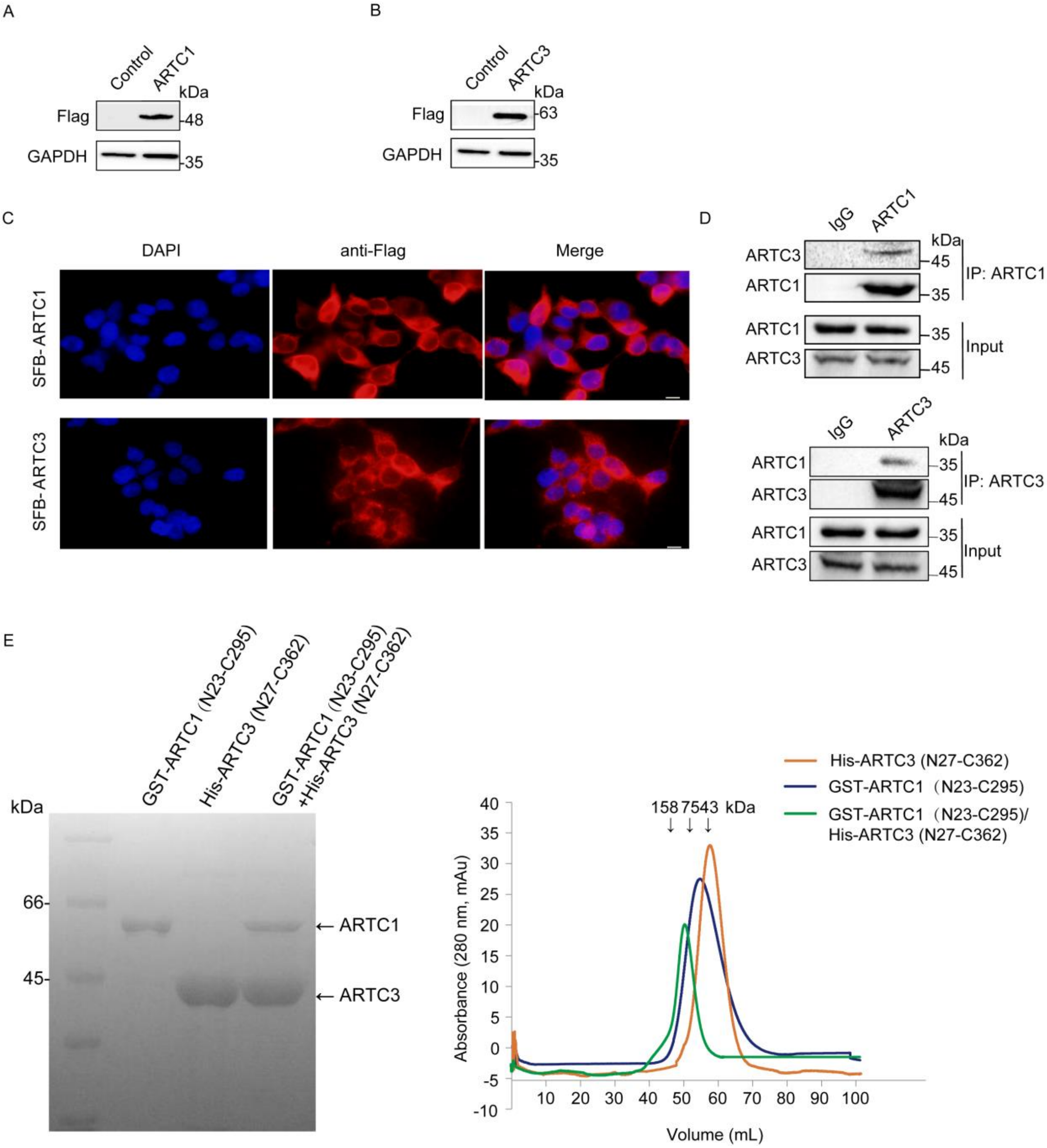

Supplementary Figure S2 Detection of the efficiency of hARTC1- and hARTC3- overexpressed stable cell lines and the interaction between hARTC1 and hARTC3. (**A, B**) The expressions of hARTC1 and hARTC3 in hARTC1- and hARTC3-overexpressed stable 293T cell lines were determined by western blotting (n = 3 independent experiments). (**C**) Immunofluorescence analysis of hARTC1- and hARTC3- overexpressed stable 293T cell lines (n = 3 independent experiments). (**D**) The interaction between hARTC1 and hARTC3 was analyzed in 293T cells using endogenous Co-IP (n = 3 independent experiments). (**E**) hARTC1 and hARTC3 form a stable heterodimer. Size exclusion chromatography showed that GST-ARTC1 (N23-C295) and His-ARTC3 (N27-C362) formed a heterodimer with a molecular weight of 94 kD, while the molecular weights of GST-ARTC1 (N23-C295) and His-ARTC3 (N27-C362) were 56 kD and 38 kD, respectively (right panel). The purified ARTC1 and ARTC3 were displayed in the left panel using SDS-PAGE.

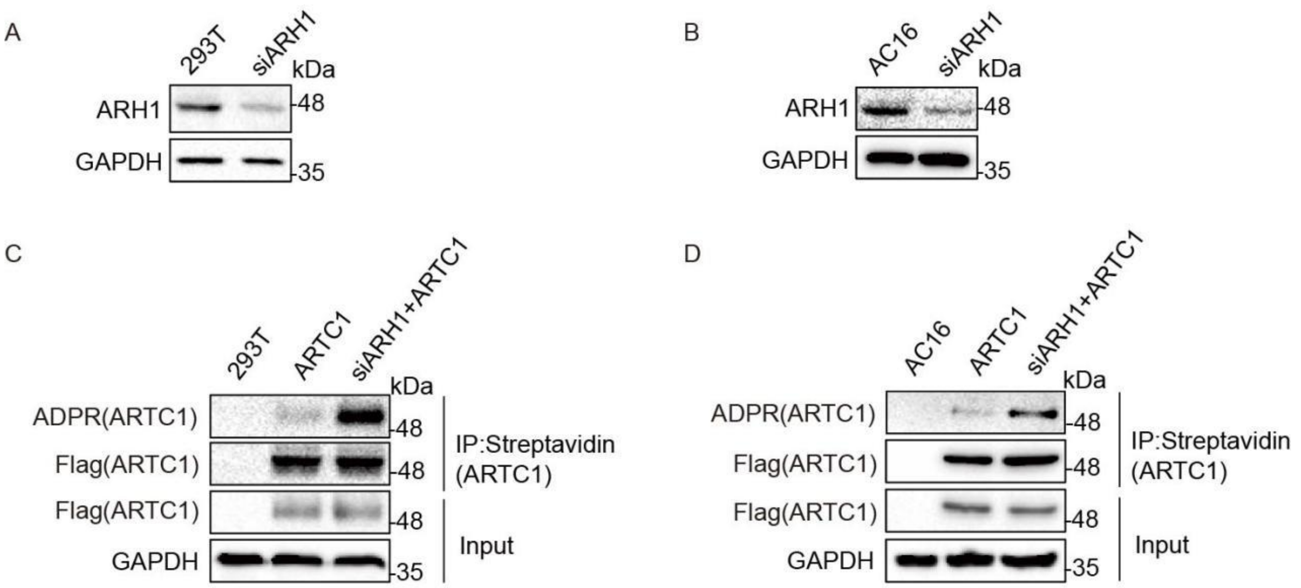

Supplementary Figure S3 Detection of the auto-modification of ARTC1 in ARH1 knockdown cells. (**A, B**) Western blotting analysis demonstrating the knockdown efficiency of siRNA-ARH1 in 293T and AC16 cells (n = 3 independent experiments). (**C, D**) Knockdown of ARH1 significantly increased the mono ADP-ribosylation of hARTC1 (n = 3 independent experiments).

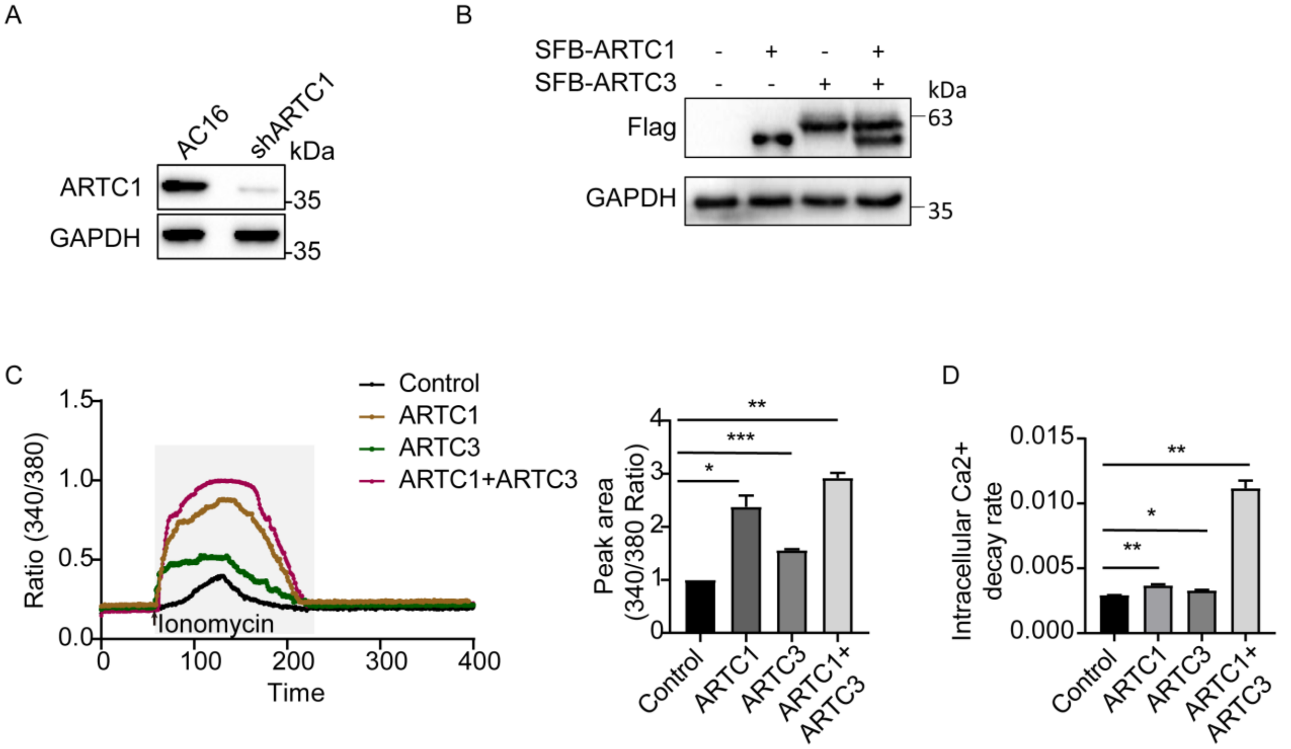

Supplementary Figure S4 ARTC3 enhances the role of ARTC1 in regulating calcium homeostasis. (**A**) Identification of stable cell lines with hARTC1 knockdown by western blot analysis (n = 3 independent experiments). (**B**) SFB-ARTC1 or SFB-ARTC3 plasmids were transfected into AC16 cells as indicated. The expressions of SFB-ARTC1 or SFB-ARTC3 were subsequently analyzed using anti-FLAG antibody by western blotting. GAPDH was used as a control (n = 3 independent experiments). (**C**) Ionomycin (1 μM)-triggered Ca^{2+} signaling in control, ARTC1-overexpressed, ARTC3-overexpressed and ARTC1 + ARTC3-co-overexpressed cells. Fura-2 (340/380 ratio) was used to measure the intracellular Ca^{2+} concentration. The histogram shows the average peak area of Ionomycin-triggered Ca^{2+} transients in each group (n = 3 independent experiments). *: $P < 0.05$, **: $P < 0.01$, ***: $P < 0.001$. (**D**) The histogram depicts the intracellular Ca^{2+} decay rate with Ionomycin-triggered Ca^{2+} transients in each group. *: $P < 0.05$, **: $P < 0.01$.

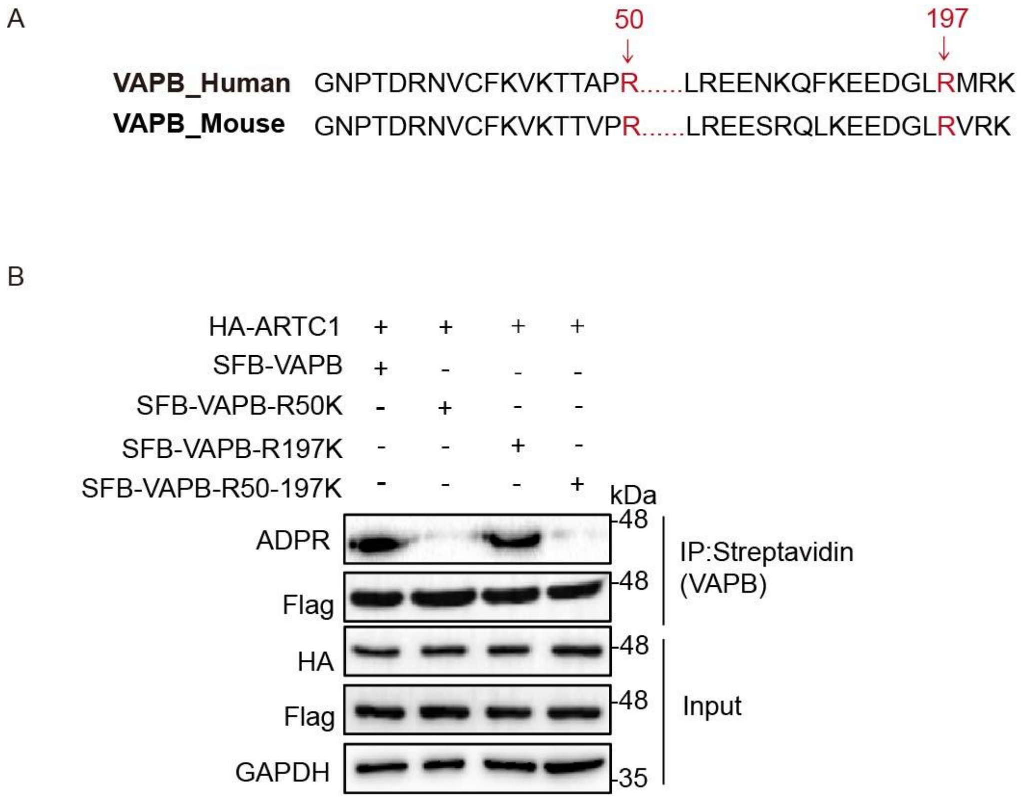

Supplementary Figure S5 Detection of ADP ribosylation sites on VAPB. (**A**) Homology alignment of mouse and human VAPB. (**B**) ADP-ribosylation of the VAPB mutant at Arg197 was not impaired compared to the control group (n = 3 independent experiments).

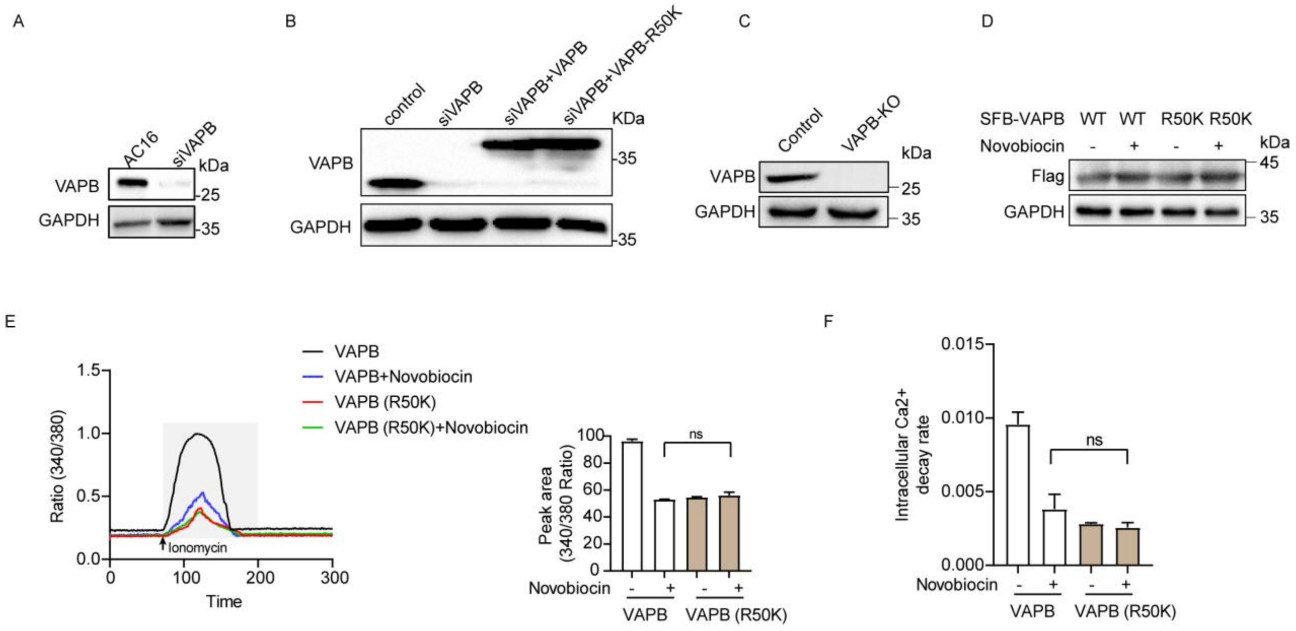

Supplementary Figure S6 Detection of VAPB-knockdown or knockout cell lines and the impact of ADP-ribosylation of VAPB on Ca^{2+} signaling. (**A**) Western blotting analysis shows a significant decrease in VAPB protein levels after siVAPB transfection in AC16 cells (n = 3 independent experiments). (**B**) AC16 cells were treated with siVAPB to knockdown endogenous VAPB. Wild-type VAPB or the R50K mutant was reintroduced into the cells. The expression of VAPB was examined by western blotting. GAPDH was used as a control (n = 3 independent experiments). (**C**) Detection of VAPB-knockout (KO) stable cell lines. Western blotting analysis of VAPB knockout efficiency in AC16 cells (n = 3 independent experiments). (**D**) Wild-type VAPB or the R50K mutant was reintroduced into the VAPB-KO cells. The expression of VAPB was examined by western blotting. GAPDH was used as the control (n = 3 independent experiments). (**E**) Intracellular Ca^{2+} levels and the Ca^{2+} decay rate in VAPB KO cells reconstituted with VAPB-WT or VAPB-R50K treated with the ARTC inhibitor novobiocin or not. The histogram shows the average peak area of ionomycin-triggered Ca^{2+} transients in each group (n = 3 independent experiments). ***: $P < 0.001$. (**F**) The histogram shows intracellular Ca^{2+} decay rate with ionomycin-triggered Ca^{2+} transients in each group of (**E**). **: $P < 0.01$.

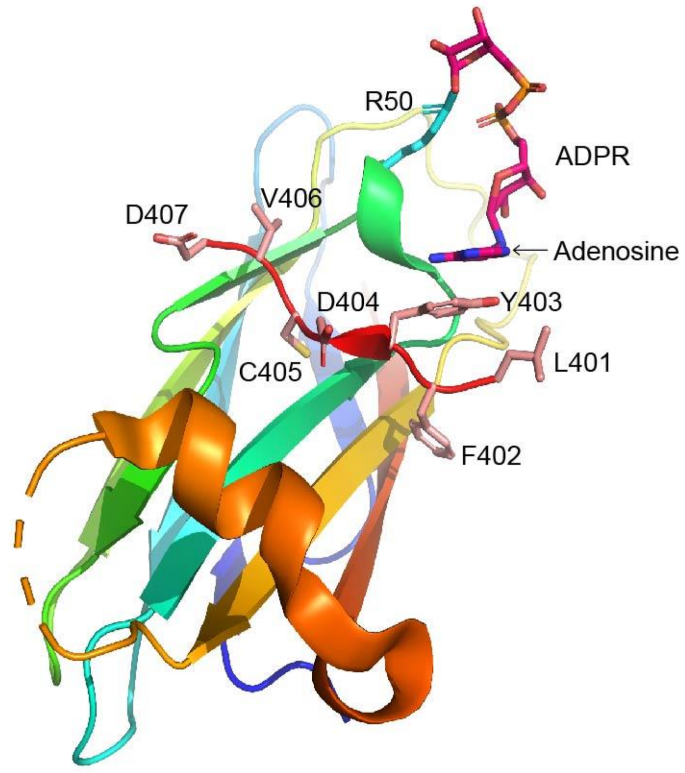

Supplementary Figure S7 Prediction of the crystal structure of interaction between VAPB (1-125 aa) and SCRN1 (401-407 aa). The ADP-ribosylation on Arg^{50} of VAPB may enhance its ability to bind SCRN1 by forming π-π interaction between the adenosine ring of ADPR and the aromatic ring of Tyr^{403}.

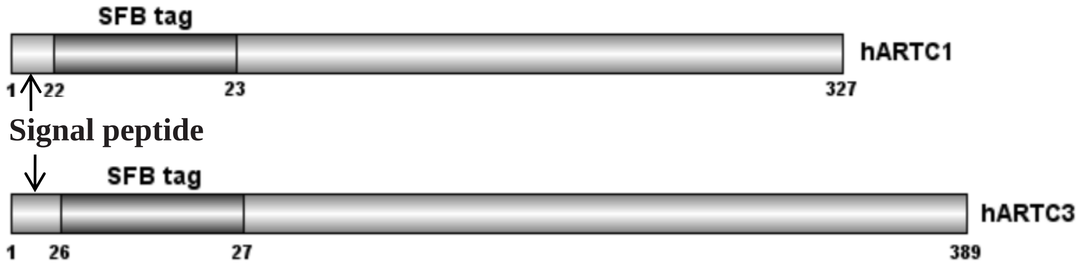

Supplementary Figure S8 The schematic for hARTC1 or hARTC3 cloned into the SFB vector. hARTC1 and hARTC3 were cloned into the SFB vector (SFB tag) with the signal peptide in their N-terminal.

Supplementary Tables

Supplementary Table S1 Relevant clinical information of the samples used in the study.

Case ID	Histology	Age at diagnosis	Gender
780751	Esophagus	71	F
737995	Liver	66	M
792337	Ovary	65	F
773364	Gallbladder	36	F
759677	Lung	62	M
769691	Thyroid	29	M
783362	Stomach	53	M
753496	Breast	67	F
780210	Testis	21	M
796358	Skeletal muscle	63	M

Supplementary Table S2 The primers used in the study.

Primer name	Sequence (5'-3')
hARTC1-qPCR-Forward	ACACCTTCTTCGGCATCTGG
hARTC1-qPCR-Reverse	TCAGCACCTCTTCCTCTCCA
hARTC3-qPCR-Forward	TCCTTGAACCCACCCAAATACC
hARTC3-qPCR-Reverse	GATGGCTTTTGGGACCTGGA
hARTC4-qPCR-Forward	ATCGACTTCGACTTCGCACC
hARTC4-qPCR-Reverse	GTGGGCTTTTTGCCACATCC
hARTC5-qPCR-Forward	TGGCTCCAGACACCTTTGAC
hARTC5-qPCR-Reverse	GGTGGGCCATTTCCTCCTTTA
hGAPDH-qPCR-Forward	GGTCGGAGTCAACGGATTTG
hGAPDH-qPCR- Reverse	ATGAGCCCCAGCCTTCTCCAT
